# A comparison of Sysmex-XN 2000 and Yumizen H2500 automated hematology analyzers

**DOI:** 10.1016/j.plabm.2020.e00186

**Published:** 2020-10-29

**Authors:** Milena Małecka, Olga Ciepiela

**Affiliations:** a Department of Laboratory Medicine, Medical University of Warsaw, Poland; b Central Laboratory of Central Clinical Hospital, University Clinical Center, Warsaw, Poland

**Keywords:** Sysmex XN-2000, Horiba Yumizen H2500, Complete blood count, Comparison, Automated hematology analyzer

## Abstract

A complete blood count is a highly automated laboratory test. The use of highly advanced measurement methods increases the accuracy and sensitivity of the determination of individual hematological parameters, especially in the case of white blood cells differentiation. Therefore, it is necessary to make comparative analyses, which involve performance of the analyzers used in daily work. The aim of the study was to indicate whether the results obtained using two compared analyzers show significant differences.

**Materials and methods:**

In this study a comparative analysis of 241 peripheral blood samples from adult patients was performed. The complete blood count results were obtained using two automated hematology analyzers: Sysmex XN-2000 and Horiba Yumizen H2500. The Passing-Bablok regression method and Bland- Altman analysis were also used to evaluate the results received for both analyzers.

**Results:**

Statistically significant differences were found for four hematological parameters: eosinophil count, immature granulocytes, mean corpuscular hemoglobin concentration (MCHC) and platelet distribution width (PDW). The P value for MCHC was 0.01. Sysmex XN-2000 and Horiba Yumizen H2500 also showed disagreement in plate platelet distribution width (PDW) (P ​= ​0.04). For other parameters both analyzers showed good agreement.

**Conclusion:**

Based on the results of the study, it was shown that there are significant differences in the measurements of hematological parameters between compared analyzers.

## 1 Introduction

Complete blood count (CBC) is performed using automated hematology analyzers, which performance and functions are constantly improving. Currently, when evaluating CBC with leukocyte differentiation (CBC+5-DIFF), it is possible to determine about thirty basic and sixteen optional hematological parameters. The increasing number of parameters affects the reliability and usefulness of this laboratory test in the process of clinical interpretation.

It is important that the results of laboratory tests obtained using automated analyzers, which are currently able to perform more than 1000 tests per day, are comparable, repeatable and reliable regardless of the analyzer used and the laboratory in which the analysis was performed. The advanced measurement methods used in automated hematological analyzers translate to the quality of obtained laboratory results. The results are characterized by increased accuracy and sensitivity, especially in case of leukocyte differentiation. To this day, however, microscopic smear is considered to be the most reliable reference method used to assess the number and morphology of peripheral blood cells. The assessment of blood smear is performed by an experienced medical laboratory scientist. The interpretation of the blood smears’ results depends on the number of cells counted under the microscope [[1], [2], [3], [4]].

There are many comparative analyses in the literature in which the performance of automated hematology analyzers was evaluated. In this study two hematological analyzers were compared for the first time: Sysmex XN-2000 and Horiba Yumizen H2500. The results of our study indicate that there are significant differences in the determination of peripheral blood samples’ parameters [5].

## 2 Materials and methods

### 2.1 Specimens

To perform the comparative analysis, 241 samples of whole blood at a volume of 2 ​ml each, collected to tubes containing K_3_EDTA anticoagulant (Medlab, Raszyn, Poland), were taken from adult patients of both sexes aged 19–94 years. All samples came from patients who underwent hematological examination in the Central Laboratory of the University Clinical Centre of Warsaw Medical University. The evaluation was performed using the Sysmex XN-2000 and Yumizen H2500 analyzers. Samples were stored in room temperature before additional analysis. Complementary analyses were performed within 20 ​min after the completion of routine tests.

Samples of peripheral blood came from both healthy patients and those suffering from various pathologies. Pathological samples came from patients with hematological disorders (anemia of different etiology (n ​= ​58), leukemia (n ​= ​25), lymphoma (n ​= ​15), thrombocytosis (n ​= ​15), thrombocytopenia (n ​= ​10)), cancers (lung (n ​= ​10), kidney (n ​= ​10) and liver cancer (n ​= ​10)), kidney diseases (nephrotic syndrome) (n ​= ​20), diabetes (n ​= ​18), acute and chronic inflammations (n ​= ​40) and endocrine disorders (n ​= ​10). The included subjects were tested either in the inpatient or outpatient departments. Results for comparative analysis were collected over a period of four months. Samples selected for testing were randomly selected. Samples with flags were excluded from the study.

The number of patient’s samples analyzed using each automated analyzer was the same (Table 1). To perform comparative analysis of hematological parameters, complete blood count with leukocyte differentiation (CBC+ 5-DIFF) and a complete blood count (CBC) without leukocyte differentiation were used.

**Table 1:** The number of samples analyzed for Sysmex XN-2000 and YUMIZEN H2500.

Parameter	Number of samples
WBC [10^9^/L]	241
Neutrophils [10^9^/L ]	223
Lymphocytes [10^9^/L ]	223
Monocytes [10^9^/L ]	223
Eosinophils [10^9^/L ]	223
Immature granulocytes [10^9^/L ]	223
RBC [10^12^/L], HGB [g/L], HCT [%], MCV [fL], MCH [fmol], MCHC [mmol/L]	241
RDW-CV [%]	221
PLT [10^9^/L ]	241
MPV [fL], PCT [%], PDW [fl]	204

The presented analysis is a retrospective study – all samples were analyzed twice due to diagnostic requirements. A study stays with agreement with the rules of Bioethical Committee at Medical University of Warsaw.

The source of difference in number of samples for each parameter was that some of the samples were used to perform complete blood count (CBC), whereas complete blood count with differential (CBC ​+ ​DIFF) was performed using the others.

### 2.2 Instruments

The automated hematology analyzers compared in the study were run according to standard operating procedures and controlled using control materials for each analyzer once a day according to the intra-laboratory control procedure. The ambient temperature in the room where the analyzers are located requires stabilization and daily control in the range of 15–30 ​°C. Also, the reagents used for both analyzers should be stored at room temperature. Control material for both the Sysmex XN-2000 and Horiba Yumizen H2500 Analyzer should be stored in the refrigerator. The room requires 100–240 ​V of power for the Sysmex XN-2000 and Horiba Yumizen H2500 analyzers. The power consumption for the Horiba Yumizen H2500 is 200 ​V ​A, while that of the second analyzer is 270 ​V ​A or less. The noise level generated by the analyzers is 60 ​dB for the Sysmex XN-2000, and <57 ​dB for the Horiba Yumizen H2500. The dimensions of the Horiba Yumizen H2500–73 ​× ​83 ​× ​65 (HxWxD), while the Sysmex XN-2000–28 ​× ​40 ​× ​35.5 (HxWxD). The calibration of the analyzers is performed by the service installing the analyzer during the installation of the analyzers and after the control service. The calibration process is performed to establish the precision and accuracy of the analyzer using the appropriate reagents. When performing the calibration, the aim is to achieve results for each parameter close to the known target values and ranges recommended by the analyzer producents Table 2. The coefficient of variation and the percentage difference must be within the ranges specified for each parameter. On the other hand, the quality control of the determinations performed on the analyzers enables the monitoring of determinations on a sample (control material) with known hematological parameters, which are produced by the company that made the analyzer.

**Table 2:** Detection limit of Yumizen H2500 and Sysmex XN-2000.

Parameter [Units]	Detection limit of Yumizen H2500	Detection limit of Sysmex XN-2000
WBC [10^9^/L]	0.000–300	0.000–440
RBC [10^12^/L]	0.0–8.8	0.00–8.60
HGB [g/L]	0.0–24.5	0.0–26.0
HCT [%]	0.0–68.8	0.0–75.0
PLT [10^9^/L ]	0.0–5000	0.0–5000

In-laboratory control using control materials DIFFTROL L, N and H for Yumizen H2500 and XN-CHECK L1, L2, L3 for XN-2000 respectively is performed daily. After each calibration related to maintenance of the analyzers and after replacement of the control series, an in-laboratory control is performed. An in-laboratory repeatability control is also performed between the analyzers using material from one patient (whole blood collected for EDTA). Both analyzers are used for routine tests carried out in the Central Laboratory of the University Clinical Centre of the Medical University of Warsaw.

The Sysmex XN-2000 (Sysmex, Kobe, Japan) using the following methods of fluorescent flow to determine all tested hematological parameters: cytometry, impedance and optical cytometry. Impedance method to count red blood cells (RBC) and platelets, and has an optical measurement channel for reticulocytes in which platelet count can also be measured. In addition to the impedance method, RBC and PLT are determined using hydrodynamic focusing methods. The analyzer uses the technology of fluorescent flow cytometry to determine the white blood cell count (WBC) in all measurement channels. Fluorescent flow cytometry is based on scattered laser light (front and side). The inspired volume of test material in this analyzer is 88 ​μl in all modes. The capacity of the analyzer is over 100 samples per hour. Using the Sysmex XN-2000 analyzer we can determine 28 basic diagnostic parameters, which are always standard components of the device (CBC ​+ ​DIFF and NRBC). There are also 16 optional diagnostic parameters that can be marked in a sample using this analyzer. The Sysmex XN-2000 also has a measurement channel in which immature granulocytes are counted.

The Yumizen H2500 (Horiba, Kyoto, Japan) is a quantitative, multi-parameter, automatic hematological analyzer. It uses the double hydrodynamic focusing method, which includes measurement of polychromatic light absorbance and the impedance method. The analyzer automatically mixes the samples at 360 ​°C, which ensures perfect homogeneity. The analyzer capacity is 120 samples per hour for CBC-DIFF. The sample volume needed for analysis is 110 ​μl. The analyzer allows the measurement of up to 55 parameters.

PDW is a parameter calculated on the basis of the PLT histogram measured using the impedance method by both analyzers. The measurements of the number of immature granulocytes and eosinophils are performed by the XN-2000 analyzer using the fluorescence flow cytometry method, while the second analyzer - Yumizen H2500 uses the impedance method in addition to the flow cytometry method to count these cells.

### 2.3 Statistical analysis

When analyzing the results of the study, the following quantitative parameters were compared first: RBC, HGB, MCV, HCT, PLT, WBC (measured directly), as well as PDW and MCHC (calculated). A comparative analysis was also performed for the results obtained for leukocyte differentiation (percentage and absolute number of neutrophils, lymphocytes, eosinophils, basophils and monocytes).

Statistical analysis was performed in Microsoft Office Excel (Microsoft Corporation, Remond, WA) and MedCalc Software (Ostend, Belgium). The degree of compatibility between the parameters determined using the two automatic analyzers was evaluated using a non-parametric statistical regression test, Passing-Bablok [6] and Bland-Altman analysis. A value of P ​< ​0.05 was considered statistically significant for each parameter determined.

## 3 RESULTS

Reliable leukocyte differentiation results were obtained for blood samples in which the number of white blood cells perceived using an automatic hematological analyzer was within the detection limits for both analyzers.

Statistical analysis using the Passing-Bablok regression method showed statistically significant differences between the analyzers compared in several hematological parameters. Table 3 presents exact regression equations and 95% confidence intervals for intercept and slope obtained in the study of all arrangements.

**Table 3:** A passing-bablok regression analysis for sysmex XN-2000 and YUMIZEN H2500 comparison.

Sysmex XN-2000 and YUMIZEN H2500		
Parameter	Correlation coefficient (r)	Significance level (P value)
**WBC**	0.991	P ​= ​0.29
**Neutrophils**	0.947	P ​= ​0.42
**Lymphocytes**	0.887	P ​= ​0.33
**Monocytes**	0.851	P ​= ​0.16
**Eosinophils**	0.826	P ​< ​0.01
**Immature granulocyte**	0.796	P ​< ​0.01
**RBC**	0.987	P ​= ​0.68
**Hemoglobin**	0.994	P ​= ​0.46
**Hematocrit**	0.962	P ​= ​0.57
**MCV**	0.860	P ​= ​0.29
**MCH**	0.960	P ​= ​0.17
**MCHC**	0.392	P ​= ​0.01
**RDW - CV**	0.782	P ​= ​0.85
**PLT**	0.993	P ​= ​0.48
**MPV**	0.929	P ​= ​0.32
**PCT**	0.990	P ​= ​0.28
**PDW**	0.931	P ​= ​0.04

Unless the 95% confidence interval of the intercept include 0, there are systematic errors in both instruments. The slope is a measure of the difference in the ratio between the two instruments. The 95% confidence interval for the slope does not contain 1 and there is at least a proportional difference between the two methods.

To assess the difference in blood count measurements obtained using the Yumizen H500 and Sysmex HN 2000 analyzers, Bland and Altman methodology was used. According to Bland and Altman, the results can be considered fully comparable if 95% of the differences between the results of pairs of measurements are in the range: average difference ±1.96 SD. The percentage of measurements outside this range is called the Bland and Altman coefficient. If the Bland and Altman coefficient is <5%, then the measurement methods are characterized by high comparability of results. Correlation coefficient (r) and significance level (*P*-value) were put in Table 3.

**Fig. 1:**
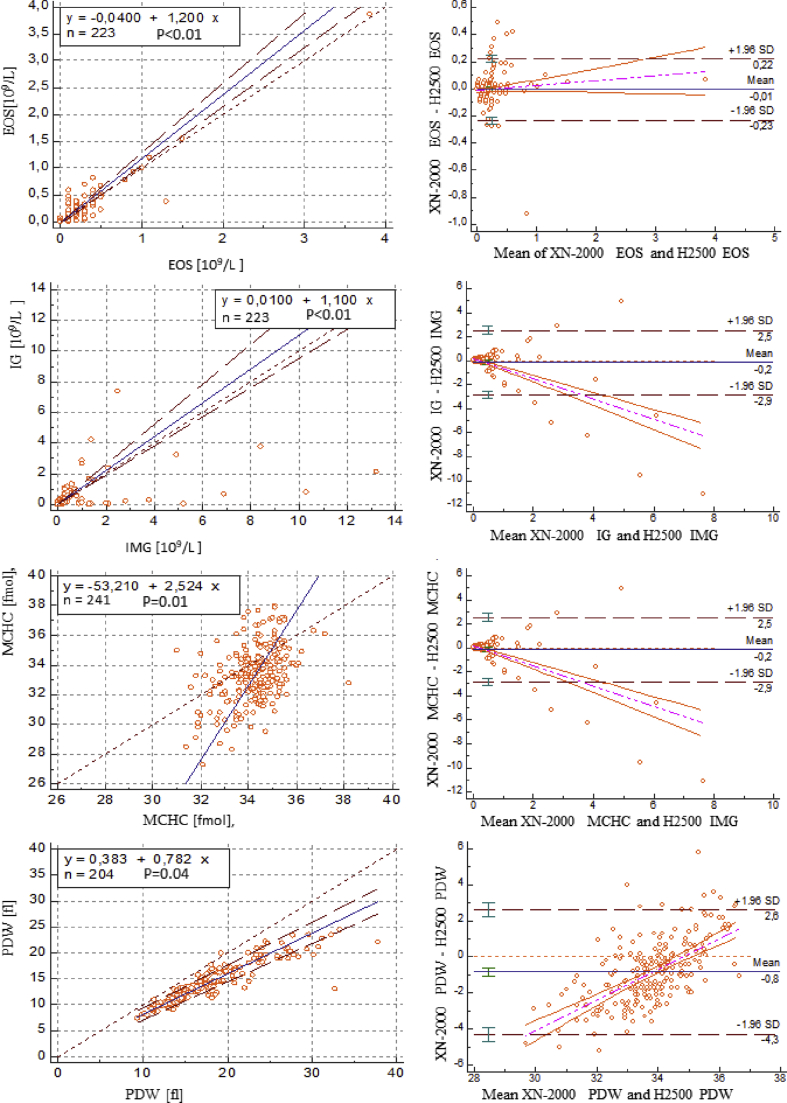
A Passing- Bablok regression and Bland-Altman Analysis for SYSMEX XN-2000 and YUMIZEN H2500 comparison. Graphs for hematological parameters showed statistically significant differences between the analyzers compared.

Bland Altman plot on Fig. 1 describe agreement between two measurements. The scatter plot XY, in which the Y axis shows the difference between the two paired measurements of the two compared analysers and the X axis represents the average of these measures.

In the comparative analysis, both parameters (PDW and MCHC) displayed statistically significant differences.

Comparison of the Sysmex XN-2000 vs. Horiba Yumizen H2500 disagreement was found for immature granulocytes and eosinophils count (Table 3) (P ​= ​0.01). and in mean hemoglobin concentration in red blood cells (MCHC). This parameter was chosen as an example of statistical significance. The P value for MCHC, which showed comparable results in XN-2000 and Horiba Yumizen H2500, was 0.01. Sysmex XN-2000 and Horiba Yumizen H2500 also showed disagreement in plate platelet distribution width (PDW) (P ​= ​0.04).

The comparative study showed the agreement between measurements of complete blood count parameters such as leukocyte count (WBC), red blood cell count (RBC), hemoglobin concentration (HGB), hematocrit (HCT), mean corpuscular volume (MCV), mean corpuscular hemoglobin (mean cell hemoglobin, MCH), red blood cell distribution width (RDW), platelet count (PLT), mean platelet volume (MPV), and plateletcrit (PCT).

No parameter was obtained for which the result would have an intercept of 1.0 or slope of 0.

## 4 Discussion

We found that despite satisfactory agreement between most analyzed parameters, the Sysmex XN-2000 and Yumizen H2500 are not fully interchangeable in terms of extended CBC analysis.

The different techniques used in the two analyzers may be the source of the variability in results. The Sysmex XN-2000 uses flow cytometry, hydrodynamic focusing method, impedance, non-fluorescent dye staining and electrical conductivity. The Horiba Yumizen H2500 analyzer differentiates the cells using flow cytometry, double hydrodynamic focusing and impedance method. Both analyzers apply flow cytometry methods, although using different reagents for red blood cell lysis and white blood cell differentiation [7].

Based on the results obtained, there are no significant differences in measurements between the Sysmex XN-2000 and Horiba Yumizen H2500 for the following complete blood count parameters: RBC, HGB, HCT, MCV, MCH, RDW, PLT, MPV and PCT. However, significant differences and wide variability of results were discovered for the following hematological parameters: eosinophil (EOS) count, immature granulocytes (IG, IMG), mean cell hemoglobin concentration (MCHC) and platelet distribution width (PDW). Even though the parameters that did not show agreement between the tested analyzers are not crucial for the determination of a patient’s condition, they might be used for patient monitoring during treatment (especially IG or EOS), or might be important for differentiating various types of anemia. Moreover MCHC is the optimal parameter for assessing intralaboratory [8] control – thus variation of its value between the tested analyzers could be misleading for laboratory personnel who perform daily maintenance.

In this analysis, results of white blood cell, red blood cell and platelet counts were consistent between the two studied automated analyzers. Incompatibility in measuring white blood cell counts have been demonstrated in studies of Meintker et al. [8]. In their benchmarking studies involving Abbot Sapphire, Siemens Advia 120, Beckman Coulter DxH 800 and Sysmex XE-2100 analyzers, showed a good correlation for the following parameters: leukocyte, monocyte, eosinophil and basophil counts, platelet and red blood cell counts [8]. Monocyte count agreement was high between all compared automated analyzers. The analyses of Grillone et al. Meintker et al. and Tan et al. showed a repeatable correlation in monocyte count by different automated analyzers [1,8,9]. To conclude the study of leukocyte count, our results are comparable with others published up to date which shows high levels of agreement.

Research done by Pérez et al. compared the Sysmex XN-2000 and Sysmex XE-5000 automated hematology analyzers. This analysis included the results of peripheral blood tests performed on 206 patient samples analyzed for the measurement of red blood cell parameters, platelet, leukocytes counts. Statistically significant differences were observed for immature granulocytes and erythroblasts. Perez I. et al.‘s results as well as our analysis showed significant differences in the measurements of immature granulocytes between different analyzers. Perez et al.‘s results also showed that the precision, accuracy and reproducibility of the analyzers were satisfactory and demonstrated reliability. The presented research provides relevant information on the measurement of immature granulocytes. As in Perez et al.‘s studies, our analysis showed significant differences in results between analyzers [10].

Platelet count agreement was high between both studied analyzers. The received results can be used interchangeably. Our results also give satisfactory reproducibility results in other comparative studies [8].

The current study showed no significant differences in measurements of red blood cell count (RBC), mean corpuscular volume (MCV) and mean corpuscular hemoglobin [mean cell hemoglobin (MCH)]. Lippi et al. [11] and Ciepiela et al. [5] reported low agreement in red blood cell count, while in our investigation, substantially higher agreement between the two hematology analyzers was observed. Lippi et al. showed high agreement in measurements of mean corpuscular volume (MCV) between the compared analyzers. In the current study we found no significant differences between the Sysmex XN-2000 and Yumizen H2500. Compared to Lippi et al. who reported limited comparability of RDW, the current study presented high agreement in RDW measurements among the analyzers used for comparison.

Our research did not show any correlation between monocyte count results, as opposed to the research by Nguyen VTP et al. [12]. A comparative study of Sysmex XN-2000 i Advia 2120i analyzers conducted by Nguyen VTP et al. showed compliance between hematological parameters’ results beside monocyte count. A higher number of monocytes was observed in results obtained with Sysmex XN-2000 analyzer in comparison to Advia 2120i.

Research by Hotton et al. which compared the results obtained using three different automated hematology analyzers (DxH-800, XN-2000 and Cell-Dyn Sapphire), showed a relevant correlation between the results for four parameters: hemoglobin (HGB), mean cell volume (MCV), platelets (PLT) and white blood count (WBC). A relevant difference was discovered only in PLT by the Bland-Altman diagrams and may have been due to the different measuring methods used for this hematological parameter [2]. The Cell-Dyn Sapphire analyzer uses an optic method of counting platelets, whereas DxH-800 and XN-2000 use an impendence method. Hotton et al. did not show any relevant correlations between the IG parameters obtained from different analyzers.

The source of the variability, as shown in our research, may have been a different method used by the analyzers to count leukocytes. The performance of XN 2000 is based on fluorescent flow cytometry, which is used to differentiate leukocytes and immature cells, whereas Horiba Yumizen H2500 counts leukocytes using impedance variation measurement and absorbency. The MCHC parameter is calculated on the basis of hematocrit and hemoglobin concentration. Contains the PDW parameter determined on the basis of the PLT histogram. In the comparative analysis, both parameters related statistically significant differences. The Horiba Yumizen H2500 analyzer utilizes the impedance method, whereas the XN 2000 uses the impedance method with hydrodynamic focusing to count RBC, HCT and HGB (SLS-HGB method), based on which the MCHC parameter is determined.

No comparative analysis of results obtained using the Yumizen H2500 series automated analyzer manufactured by Horiba or other analyzers available on the market are available.

On the basis of the presented results there are significant differences in the measurements of hematological parameters between the analyzers in question. The study did not analyse bias of the Sysmex XN-2000 and Horiba Yumizen H2500, but regardless, the results obtained from any of the analyzers in question cannot be replaced by those obtained from another.

In conclusion, due to the existence of variability between the results obtained from different hematology analyzers, it is recommended that patient samples be analyzed in the same laboratory using the same analyzer, in order to avoid obtaining divergent results that could be misinterpreted. Incorrect interpretation may affect further clinical management and be a source of uncertainty.

## Author statement

Author: O. Ciepiela: Conceptualization, Resources, Writing - Original Draft, Writing - Review & Editing.

Author: M. Małecka: Formal analysis, Investigation Writing - Original Draft, Writing - Review & Editing.

## Declaration of comprting interest

None.
